# Novel Adipokines and Bone Metabolism

**DOI:** 10.1155/2013/895045

**Published:** 2013-02-04

**Authors:** Yuan Liu, Cheng-Yuan Song, Shan-Shan Wu, Qiu-Hua Liang, Ling-Qing Yuan, Er-Yuan Liao

**Affiliations:** ^1^Institute of Metabolism and Endocrinology, Second Xiangya Hospital, Central South University, Changsha 410011, Hunan, China; ^2^Geriatric Department, Second Xiangya Hospital, Central South University, Changsha 410011, Hunan, China; ^3^Neurology Department, Xiangya Hospital, Central South University, Changsha 410008, Hunan, China

## Abstract

Osteoporosis is a serious social issue nowadays. Both the high morbidity and its common complication osteoporotic fracture load a heavy burden on the whole society. The adipose tissue is the biggest endocrinology organ that has a different function on the bone. The adipocytes are differentiated from the same cell lineage with osteoblast, and they can secrete multiple adipokines with various functions on bone remolding. Recently, several novel adipokines have been identified and investigated thoroughly. In this paper, we would like to highlight the complicated relation between the bone metabolism and the novel adipokines, and it may provide us with a new target for prediction and treatment of osteoporosis.

## 1. Introduction

Bone is the principal structural connective tissue that supports the whole body and protects the delicate organs inside. It also serves as an endocrine organ that plays a pivotal role in the regulation of the mineral homeostasis. Being modulated by multiple factors, the bone tissue keeps continuously undergoing biological processes of regeneration by osteoblast and absorption by osteoclast. Respectively, osteoblasts originate from the bone marrow mesenchymal stem cells (MSCs) which are a series of polypotential cells while osteoclasts are raised from the hematopoietic lineage mainly located in the blood and the bone marrow. The normal differentiation, proliferation and maturation of these two cells are the prerequisite of bone homeostasis. Actually, in a healthy bone, there is an elaborate balance between the number and activity of these two cells which is maintained by various systemic hormones and local factors such as the parathyroid hormone, 1,25-dihydroxy vitamin D3, and loading effect. This balance promises the normal bone turn over and remolding, and once the balance is broken down and the absorption overrides the regeneration, osteoporosis occurs. 

 Fat is the biggest endocrine organ in human body, it severs as an important part in the energy restoration and nutrition metabolism. It also has a complex connection with the bone. A common phenomenon is that, with aging, the fat tissue in the bone marrow increased while the bone mass decreased. Actually, both the adipocyte and osteoblast are originated from the same cell lineage: MSCs. The differentiation trend to preadipocytes or preosteoblasts is competitive and being inhibited by each other. However, growing evidence suggests the fat tissue may have a more complicated effect on the bone tissue which is realized by adipokines. Previously, several studies have demonstrated that TNF-*α* and IL-6 were involved in bone metabolism through mutiple pathways like the TGF-*β*, RANKL/RANK pathway, STATs, and so forth [1–3]. Recently, lots of grand new adipokines were discovered, such as adiponectin, leptin, and vaspin. These adipokines might participate in the bone metabolism through different mechanisms. Here, we get a review on the relationship between this novel adipokines and bone metabolism.

## 2. Adipokines and Bone Metabolism

Novel adipokines, including adiponectin, leptin, resistin, chemerin, omentin, vaspin, and visfatin, participate in wildly physiological and pathophysiological procedure ranging from the eating behavior modulation, fatty acid oxidation, and energy expenditure, and so forth [4]. And also the relationship between the adipokines and the bone metabolism is very close and received a highly attention in both the clinical and biological researches. 

### 2.1. Adiponectin and Bone Metabolism

Adiponectin, also known as GBP28 (gelatin-binding protein of 28 kDa) or apM1 (adipose most abundant gene transcript 1), is secreted predominantly by differentiated adipocytes and widely participated in multiple metabolic procedure including bone metabolism. 

It has a great effect on promoting the bone regeneration by affecting both of the two processes in osteogenesis: the differentiation of MSCs to preosteoblasts as well as the osteoblasts proliferation and maturation.

 MSCs are a series of pluripotency and self-renewing cell; it can competitively differentiate into osteoblast, adipose cells, and chondrocyte under different conditions. Studies showed that adiponectin could promote the osteogenic differentiation while it could inhibit the adipocyte formation. In this procedure, cyclooxygenase-2 (COX2) is a key factor. Lee and his colleagues found that, through the COX2-dependent manner, adiponectin could promote the osteogenic differentiation of the MSCs by increasing the expression of alkaline phosphatase (ALP), osteocalcin (OC), and type 1 collage (Col-I) [5]. Meanwhile Yokota found that also by the COX2-dependent manner, adiponectin could inhibit the adipose differentiation of both MSCs and cloned stromal preadipocytes [6]. To some extent, reducing the number of adipocyte could increase that of osteoblast due to the competitive relation of these two cells during differentiation. So, adiponectin could promote the MSCs osteogenic differentiation both directly and indirectly. Moreover, adiponectin stimulates the proliferation, maturation, and mineralization of osteoblasts. Kanazawa and colleague found that after 24-hour incubation with adiponectin, BrdU incorporation was significantly enhanced in MC3T3-E1 cells, this result showed a positive effect of adiponectin on proliferation [7]. Also, Huang and colleagues found that incubating with adiponectin could induce the expression of bone morphogenetic protein-2 (BMP-2) in osteoblasts by the p38 signal pathway [8]. Moreover, Oshima and colleagues found that MC3T3-E1 cells incubated with 3 *μ*g/mL recombinant adiponectin showed a dose-dependent increasing expression of Col-I, OC mRNA and ALP activities in a 12-day culture [9]. 

Besides the positive modulation on osteoblasts, adiponectin also has a negative effect on osteoclasts. In Oshima's study, they found that adiponectin inhibited the M-CSF and RANKL induced osteoclastic differentiation of both mouse macrophages and human CD14^+^ mononuclear cells and therefore suppressed the bone-resorption activity of osteoclasts. In the osteoclastic precursor RAW264 cells, adiponectin was able to inhibit the TLR4-mediated NF-*κ*B activity and the subsequent osteoclastogenesis [10]. Interestingly, Luo et al. found that the suppressed effect was time depended. The sustained release of adiponectin by matrigel controlled-release system, instead of short-term administration, had the ability to suppress the osteoclastic activity, and this effect worked in both in vitro and in vivo studies [11]. Another in vivo study showed that the increase of adiponectin expression by adenovirus could increase trabecular bone mass, accompanied with reduction in the number of osteoclasts and plasma cross-linked N-telopeptide of type I collagen (NTx) level [9]. In summary, adiponectin can increase the bone mass by promoting bone formation and impeding bone absorption.

Also, adiponectin has a promoting effect on chondrocytes. Adiponectin at 0.5 *μ*g/mL can increase chondrocyte proliferation, proteoglycan synthesis, and matrix mineralization which were reflected by the upregulation of type II collagen, aggrecan, Runx2, and ALP activity [12].

Adiponectin has a great impact on promoting bone mass in vitro; however, during the clinical epidemiological survey, the results are contradictory as whether serum adiponectin concentration and bone mineral density (BMD) are associated. Several researches showed that the serum adiponectin level had no relation with the BMD both in premenopausal and postmenopausal woman. Also they found that serum adiponectin levels were not significantly different between the patients with osteoporotic fractures and nonosteoporotic fractures [13–16]. However, most of the researches showed a negative correlation between adiponectin and BMD [17–23]. In a META analysis including 59 studies, a great inverse correlation between adiponectin levels and BMD independent of gender and menopausal status was found. Moreover, overexpression of adiponectin can be recognized as a high-risk predictor for vertebral fractures in men [24]. 

It is a little tricky that there is a positive relationship between adiponectin and bone metabolism in the biological research while there is a negative correlation in the clinical investigation. The following reasons may account for these discrepancies. 

First, the serum adiponectin concentration is affected by multiple factors, like the age, gender, race, smoking, diabetes status, hormone levels, and so on [19, 25–28]. For instance, in healthy people, the serum adiponectin concentration is 14.2 ± 8.6 *μ*g/mL [29], but in diabetic men is 6.31 ± 4.10 *μ*g/mL and in diabetic women is 9.08 ± 7.32 *μ*g/mL [27]. Although the researches had adjusted for the age, gender, and other elements, they all have their own limitations that may influence the results.

What's more, the convincing way to study the relation between adiponectin and bone cells is measuring the adiponectin concentration in the bone marrow plasma instead of serum. The effect of the cytokines and hormones in bone marrow microenvironment exerts influence directly on MSCs and osteoblast. Several findings suggested that adiponectin had a paracrine role on bone marrow and mesenchymal progenitor cells. However, all the studies we mentioned before were talking about the serum adiponectin concentration and BMD, but the relationship between bone marrow plasma adiponectin and BMD is still not clear. We cannot simply consider that the serum adiponectin concentration is the same as that in bone marrow. In fact, in Berner's study, the adiponectin protein level in bone marrow plasma was much higher than that in serum or bone marrow cells, and the difference between these two levels diminished with aging [30]. However, Modder showed in his research that the adiponectin levels were higher in peripheral when compared to bone marrow plasma in healthy postmenopausal women [31]. Unfortunately, the relation between normal people or osteoporosis patients still needs to be elucidated. So, if we are able to measure the adiponectin in the bone marrow plasma and analyze its correlation with BMD, we may get a consistent result. Neverthless, according to the previous investigation, serum adiponectin concentration was negatively correlated with the BMD. This negative relation has profound clinical significance as serum adiponectin levels still can work as a predictor of osteoporosis in some certain population. However, further researches are still needed.

### 2.2. Leptin and Bone Metabolism

Leptin is a 16-kDa protein hormone that is primary being secreted by the white fat tissue. As early as 1994, the researchers in Rockefeller University had already found and cloned this adipokine. During the past 20 years, groundbreaking studies about leptin had been finished. 

Now we can tell various cells such as the undifferentiated bone marrow MSCs, hematopoietic cells, adipocytes, and osteoblasts, osteoclast express leptin receptor. Also, researches have already demonstrated its important role in appetite modulation, energy consumption, and body weight regulation. As early as 1999, Thomas et al. had already found that leptin could promote osteogenesis differentiation in bone marrow MSCs [32]; however, the function of leptin on bone formation is still controversial.

According to the in vitro studies and in vivo investigation, leptin acts a protective role on bone mass. On one hand, Xu et al. found that leptin could stimulate the proliferation and osteoblastic differentiation of both BMSCs and dental stem cells [33, 34]. And for the osteoblast and chondrocyte, leptin also was a promoter in the proliferation [35]. Moreover, in the fracture rat, the serum leptin is greatly increased after the injury indicated a positive relationship between the leptin and bone regeneration [36, 37]. On the other hand, incubating with leptin could decrease the osteoclastic activity of both human peripheral blood mononuclear cells (PBMCs) and murine spleen cells [38]. And during the fetal growth and development, there was a negative correlation between the concentration of serum leptin and bone resorption marker cross-linked carboxy-terminal telopeptide of type I collagen (ICTP) [39]. Moreover, van der found that, in the leptin deficient mice, the number of osteoclasts in peripheral blood was increased while cortical bone parameters were decreased in an age-related way [40]. These results all indicated that leptin might increase bone formation and decrease bone resorption with the overall effect of increasing bone mass.

However, in vivo interventional studies, the situation is much more complicated. Unlike the other adipokines which lay effect on bone only through endocrinology way, leptin affect the bone metabolism through two mechanisms: the endocrinology system and nervous system. Correspondingly, there were two administration ways to study the signaling pathway involved: subcutaneously injection and intracerebroventricular (ICV) injection.

In the subcutaneously model, studies indicated a stimulative role of leptin exerted on bone formation. Giving leptin to pregnant mice at the early stage of pregnancy could greatly increase the baby mice's ossification centers by activating the differentiation and proliferation of both chondrocyte and osteoblast [41]. Also, in the leptin receptor-deficient db/db mice, a decline in bone growth, osteoblast-lined bone perimeter and bone formation rate were observed, and those phenoma could be reversed after following subcutaneous administration of leptin [42]. Leptin also can increase the bone mineral apposition rate and bone mineral content in mice [43]. These all illuminated a positive association between the leptin and the bone formation. 

Nevertheless, in the ICV model, the effect of leptin is still unclear. Although Bartell found that culture of isolated bone marrow MSCs from the mice after ICV leptin injection showed an increase of the bone formation and BMD along with decrease of osteoclastogenesis [44], it is more widely accepted that the nervous system exerts negative effect on bone downstream of leptin administration. Ducy et al. found that the leptin inhibited bone formation through the nervous system [45]. Moreover, because leptin did not have receptors on hypothalamus, its function was mainly realized by decreasing the serotonin level. Serotonin is a neurotransmitter which is synthesized and released by brainstem, and it could bind to its receptor Htr2c and led to the activation of the sympathetic nervous system [46]. And also in Elefteriou's study, they found that the sympathetic nervous system would release noradrenaline after the administration of leptin. The noradrenaline would bind to its receptor Adrb2 (beta2-adrenergic receptors) which would lead to the production of RANKL (Receptor Activator of Nuclear Factor Kappa B Ligand) in osteoblasts and then activate the osteoclasts and decrease bone mass as the final result [47]. However, about how the nervous system works downstream of leptin is still insufficiently elucidated.

 According to 20 papers about the relation between serum leptin and BMD published during 2007–2012 [23, 48–66], 17 of them showed no correlation between this two elements after adjusted for the age, gender, or hormonal level among others. For the rest of the three papers, one paper showed a positive correlation among prepubertal girls (48 cases) [54] while another showed that leptin is inversely associated to BMD in Brazilian obese adolescents (109 cases) [61]. And the last one showed that Leptin was positively related with the whole body and femoral BMD in postmenopausal nondiabetic elderly women (63 cases) [23]. However, they are not very trustful for their limited cases and the certain group they faced. So simply from the epidemiological aspect, there is no correlation between leptin and BMD, and also leptin is not an independent predictor for fracture risk. 

In summary, the leptin exerts a rather complicated and inconsistent effect on bone metabolism from both biological and epidemical aspects, so we still need to carry more researches to unveil the truth.

### 2.3. Resisten and Bone Metabolism

Resisten, also known as adipose tissue-specific secretory factor (ADSF) or C/EBP-epsilon-regulated myeloid-specific secreted cysteine-rich protein (XCP1), was initially identified as a factor produced by adipocytes and named for its ability to induce insulin resistance [67]. Although it is widely expressed in myocytes, hepatocytes, adipocytes, MSCs, preosteoclasts, and osteoblasts, the expression level is much higher in bone marrow compared to other tissues [68]. That indicates that the resistin may play an important role in the bone metabolism. Actually, according to the recent researches, the answer is positive. 

It can stimulate the proliferation of osteoblasts through the PKC and PKA-dependent way [69]. However, on the maturation of the osteoblasts, resisten only has a weak effect. It only can mildly increase the mRNA expressions of Col-I after the administration of 7 days whereas those of Runx2, osterix, OC, and ALP were no significant difference from untreated cells. Although its effect on osteoblasts is weak, resistin has a strong effect on the osteoclastogenesis by greatly increasing the number of osteoclast and activating the NF-*κ*B promoter [69]. So, resisten is a negative factor for the bone mass which is consistent with the epidemiological studies that the serum resisten concentration is negatively correlated with the whole body BMD in Chinese men and postmenopausal women as well as the lumbar spine BMD in male adult [17, 52].

### 2.4. Visfatin and Bone Metabolism

Visfatin, which is also known as pre-B cell colony-enhancing factor (PBEF) or nicotinamide phosphoric acid RNA transferase (Nampt), is isolated as a novel adipokine preferentially expressed in visceral adipose tissue when compared to subcutaneous fat. Researchers also found a high expression of visfatin in bone marrow plasma, and this finding demonstrated a potential possibility that visfatin might modulate bone metabolism [70]. Later, this assumption was proved by our previous study: Visfatin could stimulate human osteoblast proliferation and increase the expression of osteogenic marker Col-I in a dose- and time-dependent manner. And it also could cause an increase in mineralization of osteoblasts [71]. On the other side, treatment with visfatin inhibitor FK866 reduced osteogenesis by reducing ALP activity and bone nodule formation in primary cultured BMSC [72]. Moreover, visfatin might be a negative regulator in osteoclastogenesis. Visfatin suppresses the differentiation of CD14^+^ monocytes into multinucleated TRAP^+^ osteoclasts dose dependently [73]. Nevertheless, there is no relation with bone mineral density that was found during the clinical investigation [52, 63, 74, 75]. However, in Anastasilakis' research, they found that visfatin could be a predictor of acute phase reaction (APR) caused by intravenous zoledronate treatment, for instance, lower visfatin levels at baseline accompanied with higher risk for APR [76]. This founding is very profound for the clinical usage of diphosphate.

### 2.5. Vaspin and Bone Metabolism

Vaspin was a recently identified adipokine, playing a protective role in many metabolic diseases, ranging from diabetes mellitus to atherosclerosis. The researches about vaspin were mainly focused on the insulin resistance [77], hepatitis disease [78, 79], and cardiovascular disease [80, 81]. Presently, our previous study showed that the vaspin could inhibit the apoptosis of the osteoblasts [82]. However, no report about its function in osteogenesis, osteoclastogenesis as well as the relation with BMD was published. 

### 2.6. Chemerin and Bone Metabolism

Chemerin is also a kind of adipokines that can modulate the adipogenesis and osteogenesis. Its modulation function mainly stands on the adipogenesis aspect. Expression and secretion of chemerin dramatically increased during adipocytes differentiation. And knock-down chemerin or chemerin like receptor 1 (CMKLR1) expression could abrogate adipocytes differentiation and increased osteoblast marker gene expression and mineralization after osteoblastic stimulations [83]. So, chemerin actually is a negative regulator during osteogenesis for its facilitating role in adipogenesis.

### 2.7. Omentin-1 and Bone Metabolism

Omentin-1 is a novel 34 kDa adipokine with a great function on the glucose metabolism and insulin resistance, and it is recognized as a biomarker of metabolic disorders [84, 85]. Also, it has an important function on bone metabolism. 

Our previous study found that omentin-1 inhibited osteoblastic differentiation while it had no direct effect on osteoclastic differentiation in vitro. What's more, in the ovary ectomized mice, overexpression of omentin-1 by adenovirus led to a decline of the serum OC, tartrate-resistant acid phosphatase-5b, and RANKL/OPG ratios, and a lower BMD was observed too [86].

 Also, the negative relation was found in epidemic survey. Omentin-1 level was negatively correlated with BMD in the anorexia nervosa girls [87] and Iranian postmenopausal women [74]. However, since this conclusion was deduced from limited groups instead of large population, we still need more data to confirm this potential negative relation.

## 3. Future

Osteoporosis is now a worldwide problem that bothers a lot of patients. According to the investigation, the estimated number of people with osteoporosis is approximately 10–12 million in the United States [88]. And in Austria about 5.9% of men and 22.8% of women over 50 years old face the osteoporosis problem [89]. This situation is even more serious in developing countries. The FRACTURK study showed that the prevalence of osteoporosis at the femoral neck was 7.5% and 33.3% in men and women aged 50 years or more in Turkey [90]. And in China, the data showed that the prevalence of osteoporosis at least one site in Chinese women was 23.9 ± 13.3% in the age between 50 and 59 while the prevalence increased with aging [91]. So, osteoporosis becomes a serious social problem and called for more attention. 

Current drugs admitted by the U.S. Food and Drug Administration (FDA) for osteoporotic treatment include Alendronate, Risedronate, Zoledronic acid, and Teriparatide. The first three drugs belong to the diphosphonate, the first line therapy of osteoporosis. The function of diphosphonate is mainly focused on the inhibition of the osteoclast and decrease of the bone resorption. It can only slow down the bone decline instead of reversing it. And Teriparatide, the human recombinant parathyroid hormone 1–34, has been demonstrated to be a new and promising way for severe osteoporosis treatment for its function on bone regeneration. It can increase the BMD, decrease the fracture risk, and improve the clinical symptoms such as the back pain in osteoporosis patients [92–94]. So, it is recommended for patients with severe osteoporosis. According to the study, Diphosphonate reduced vertebral fractures by 40% to 70% and nonvertebral fractures by 20% to 35% while Teriparatide reduced vertebral fractures 65% and nonvertebral fractures 53% [95].

What's more, there are some other second line drugs for osteoporosis treatment such as selective estrogen receptor modulators (SERMs), calcitonin and denosumab. Although we have plenty of drugs in using, the situation of osteoporosis treatment that is not optimistic. According to the investigation, 15 million osteoporosis patients in Japan were bothered by osteoporosis while only 20% of them were under treatment [96]. Situation in Switzerland is even worse: every other woman and every fifth man aged 50 years or older will suffer an osteoporotic fracture during her or his remaining lifetime [97]. The low under treatment rate and high osteoporotic fracture rate are warning us that there is a long way for osteoporosis prevention and treatment.

Fat tissue, as the biggest endocrinology organ, draws an important impact on the bone metabolism not only by the loading effect but also the adipokines. Those adipokines form a complex network to regulate the bone regeneration and resorption as we mentioned before (see in Table 1). Most of them have a positive effect on bone formation while some of them could also inhibit or stimulate the osteoclast activation as well. That provides us a future possible target for new osteoporosis drugs.

Moreover, during the clinical investigation, some adipokines, such as adiponectin or resisten, were found to have a positive or negative relation between the serum concentration and BMD, and this provides us with a future predictor of osteoporosis risk or osteoporotic fracture risk in some certain population. And the negative correlation between visfatin and acute phase reaction (APR) caused by intravenous zoledronate treatment is of great clinical importance too.

In summary, it has a great meaning to study the adipokine and the relation with bone metabolism. Although to date some results may be conflicting and most of the study is still in the lab, there is a promising future for the adipokines, the new therapeutic target and predictor. 

## Figures and Tables

**Table 1:** Relation between adipokines and bone metabolism.

Adipokines	Osteogenic	Osteoclastic	Relationship with BMD
Adiponectin	Promotes the MSCs osteogenesis and osteoblast maturation [5–9]	Inhibits the osteoclastogenesis and osteoclastic activity [9–11]	Negative related with the BMD; a predictor of osteoporotic fractures in certain population [11–18]

Leptin	Stimulate osteogensis both in vitro studies and subcutaneous injection animal model [19–25]	Inhibits the osteoclastogenesis and osteoclastic activity [26–31]	No relationship was found after adjust for age, gender and hormone [17, 32–50]

Resistin	Stimulates proliferation and weakly promotes the osteoblasts maturation [51]	Strongly increases the number and activity of osteoclast [51]	Negative related with certain population [11, 36]

Visfatin	Stimulates human osteoblast proliferation, maturation, and mineralization [52, 53]	Inhibits the formation of osteoclast [54]	No relationship was found [36, 47, 55, 56]

Vaspin	Inhibits the apoptosis of osteoblast [57]	No paper published	No paper published

Chemerin	Negative effect by greatly promoting the adipogenesis of the MSCs [58]	No paper published	No paper published

Omentin-1	Inhibits the osteoblastic differentiation [59]	No paper published	Not clear, need more data [56, 60]
